# The Impact of Bacterial Biofilms on End-Organ Disease and Mortality in Patients with Hematologic Malignancies Developing a Bloodstream Infection

**DOI:** 10.1128/spectrum.00550-21

**Published:** 2021-08-18

**Authors:** Enea Gino Di Domenico, Francesco Marchesi, Ilaria Cavallo, Luigi Toma, Francesca Sivori, Elena Papa, Antonio Spadea, Giuseppina Cafarella, Irene Terrenato, Grazia Prignano, Fulvia Pimpinelli, Arianna Mastrofrancesco, Giovanna D’Agosto, Elisabetta Trento, Aldo Morrone, Andrea Mengarelli, Fabrizio Ensoli

**Affiliations:** a Microbiology and Virology, San Gallicano Dermatological Institute, IRCCS, Rome, Italy; b Hematology and Stem Cell Transplant Unit, IRCCS Regina Elena National Cancer Institute, Rome, Italy; c Department of Research, Advanced Diagnostics, and Technological Innovation, Translational Research Area, IRCCS Regina Elena National Cancer Institute, Rome, Italy; d Biostatistics and Bioinformatic Unit, Scientific Direction, IRCCS Regina Elena National Cancer Institute, Rome, Italy; e Scientific Direction, IRCCS San Gallicano Institute, Rome, Italy; University of Manitoba

**Keywords:** *Escherichia coli*, *Klebsiella*, *Pseudomonas aeruginosa*, *Staphylococcus aureus*, biofilm, bloodstream infections, coagulase-negative staphylococci, microbiology, mortality, multidrug resistance

## Abstract

Bacterial bloodstream infection (BSI) represents a significant complication in hematologic patients. However, factors leading to BSI and progression to end-organ disease and death are understood only partially. The study analyzes host and microbial risk factors and assesses their impact on BSI development and mortality. A total of 96 patients with hematological malignancies and BSI were included in the study. Host-associated risk factors and all causes of mortality were analyzed by multivariable logistic regression at 30 days after BSI onset of the first neutropenic episode. The multidrug-resistant profile and biofilm production of bacterial isolates from primary BSI were included in the analysis. Median age was 60 years. The underlying diagnoses were acute leukemia (55%), lymphoma (31%), and myeloma (14%). A total of 96 bacterial isolates were isolated from BSIs. Escherichia coli was the most common isolate (29.2%). Multidrug-resistant bacteria caused 10.4% of bacteremia episodes. Weak biofilm producers (WBPs) were significantly (*P* < 0.0001) more abundant (72.2%) than strong biofilm producers (SBPs) (27.8%). Specifically, SBPs were 7.1% for E. coli, 93.7% for P. aeruginosa, 50% for K. pneumoniae, and 3.8% for coagulase-negative staphylococci. Mortality at day 30 was 8.3%, and all deaths were attributable to Gram-negative bacteria. About 22% of all BSIs were catheter-related BSIs (CRBSIs) and mostly caused by Gram-positive bacteria (79.0%). However, CRBSIs were not correlated with biofilm production levels (*P* = 0.75) and did not significantly impact the mortality rate (*P* = 0.62). Conversely, SBP bacteria were an independent risk factor (*P* = 0.018) for developing an end-organ disease. In addition, multivariate analysis indicated that SBPs (*P* = 0.013) and multidrug-resistant bacteria (*P* = 0.006) were independent risk factors associated with 30-day mortality. SBP and multidrug-resistant (MDR) bacteria caused a limited fraction of BSI in these patients. However, when present, SBPs raise the risk of end-organ disease and, together with an MDR phenotype, can independently and significantly concur at increasing the risk of death.

**IMPORTANCE** Bacterial bloodstream infection (BSI) is a significant complication in hematologic patients and is associated with high mortality rates. Despite improvements in BSI management, factors leading to sepsis are understood only partially. This study analyzes the contribution of bacterial biofilm on BSI development and mortality in patients with hematological malignancies (HMs). In this work, weak biofilm producers (WBPs) were significantly more abundant than strong biofilm producers (SBPs). However, when present, SBP bacteria raised the risk of end-organ disease in HM patients developing a BSI. Besides, SBPs, together with a multidrug-resistant (MDR) phenotype, independently and significantly concur at increasing the risk of death in HM patients. The characterization of microbial biofilms may provide key information for the diagnosis and therapeutic management of BSI and may help develop novel strategies to either eradicate or control harmful microbial biofilms.

## INTRODUCTION

Patients with hematological malignancies (HMs) are exposed to a high risk of infectious complications due to a compromised immunological status caused by either the underlying disease or the cytotoxic effects of chemotherapy or both. Bacterial bloodstream infections (BSIs) are the most common complications observed in these patients, with a prevalence of about 25% to 30% and attributable mortality rates due to septic shock of around 40% (1, 2). Neutropenic patients are susceptible particularly to high-risk bacterial infections, which may present and evolve with a fulminant progression (3). Despite improvements in BSI management, factors leading to sepsis are understood only partially and the rates of mortality remain high in patients with HMs. The increased frequency of multidrug-resistant (MDR) bacteria due, at least in part, to the high consumption of antibiotics and prolonged hospitalizations causes an additional challenge for the therapeutic management of patients with HMs (4). Mechanisms of bacterial resistance as well as other causes of poor efficacy of antimicrobial therapy in BSIs have been extensively explored (5–7). Among them, biofilm production represents a key adaptation in natural environments and human hosts. Bacteria living in biofilms show increased resistance to antimicrobial treatment and host immune defense compared with their planktonic counterparts (8). Even aggressive and long-term antibiotic treatment, based on drug resistance profiles, may show poor efficacy in eradicating biofilm infections since a minimum bactericidal concentration cannot be achieved *in vivo* without posing serious risks of adverse effects to the host (8). Furthermore, in a biofilm-related infection, planktonic cells can disseminate from the primary site of infection and spread into the bloodstream (8). This study explores putative risk factors for the development of BSIs and mortality caused by MDR and biofilm-growing bacteria in a cohort of patients with HMs.

## RESULTS

From April 2016 to April 2019, 718 patients with HMs were admitted to the Hematology and Stem Cell Transplant Unit ward. Out of these patients, 96 (13.4%) were diagnosed with a septic BSI and included in the study. The demographic and clinical characteristics are summarized in Table 1.

**TABLE 1:** Patient demographic and clinical features at enrollment^*a*^

Baseline characteristics^*b*^	*n*	%
Median age (range)	60 (20–77)	
Diagnosis		
Acute leukemia	53	55
Lymphoma	30	31
Multiple myeloma	13	14
Presence of one or more comorbidities	43	45
Treatment phase		
ASCT	31	33
Induction chemotherapy	30	31
Salvage treatment	14	15
Other chemoimmunotherapy	21	21
Disease status		
Onset	28	29
CR	35	36
PR	16	18
Relapsed/refractory	17	18
Previous documented infections	45	47
Colonization by MDR organisms	27	27
Fluoroquinolone prophylaxis	70	73
CVC	91	95
Urinary catheter	29	30
Grade ≥ 3 mucositis	35	36
Patients with ANC of <500/mcL	93	97
Median days with ANC of <500/mcL (range)	14 (2-57)	
Patients with ANC of <100/mcL	89	93
Median days with ANC of <100/mcL (range)	10 (3–52)	
Clinical outcome		
Median value of MASCC score (range)	16 (4–23)	
Septic shock	24	25
End-organ disease	26	27
Initial antimicrobial treatment failure	43	45
CRBSI	20	21
30-day attributable mortality	8	8

a Total *n* = 96.

b ASCT, autologous hematopoietic stem cell transplant; ANC, absolute neutrophil count; CR, complete remission; PR, partial remission; MDR, multidrug resistant; CVC, central venous catheter; MASCC, multinational association for supportive care in cancer index for febrile neutropenia; CRBSI, catheter-related bloodstream infection.

Gram-negative bacteria were isolated in 63.5% (*n* = 61) of the BSI cases, while Gram-positive bacteria were 36.5% (*n* = 35). Among the Gram-negative bacteria, Escherichia coli was the most common isolate (*n* = 28, 29.2%), followed by Pseudomonas aeruginosa (*n* = 16, 16.7%) Klebsiella pneumoniae (*n* = 10, 10.4%) and Acinetobacter baumannii (*n* = 3, 3.1%) (Table 2). Among the Gram-positive bacteria, coagulase-negative staphylococci (CoNS) were the most common species (*n* = 26, 27.1%) followed by Enterococcus faecalis (*n* = 3, 3.1%), Staphylococcus aureus (*n* = 2, 2.1%), and Streptococcus mitis (*n* = 2, 2.1%). MDR strains were detected in 26 cases (27%). Overall, extended-spectrum β-lactamase (ESBL)-producing *Enterobacteriaceae* were the most frequently isolated MDR strains (*n* = 14). E. coli accounted for 85.7% (*n* = 12) and K. pneumoniae for 14.3% (*n* = 2) of ESBL strains isolated. Multidrug-resistant P. aeruginosa (MDRPA) was responsible for 6 cases of BSIs, followed by carbapenem-resistant K. pneumoniae (CRKP) (*n* = 2), A. baumannii (*n* = 2), and Stenotrophomonas maltophilia (*n* = 1). Among Gram-positive bacteria, MRSA (*n* = 1) was the only MDR isolated.

**TABLE 2:** Data on bacterial isolates

Bacterial isolate	No. of organisms	%
E. coli	28	29.2
P. aeruginosa	16	16.7
K. pneumoniae	10	10.4
A. baumannii	3	3.1
M. morganii	1	1.0
*A. veronii*	1	1.0
E. cloacae	1	1.0
S. maltophilia	1	1.0
CoNS^*a*^	26	27.1
E. faecalis	3	3.1
S. aureus	2	2.1
S. mitis	2	2.1
E. faecium	1	1.0
C. striatum	1	1.0
Total	96	100

a CoNS, coagulase-negative staphylococci.

**FIG 1:**
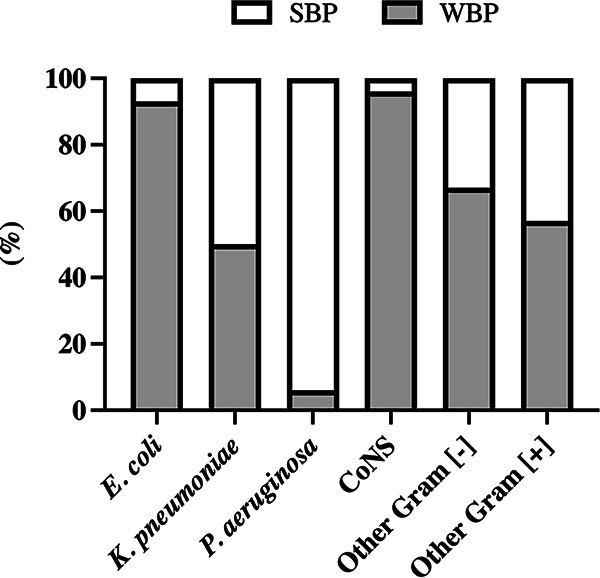
Biofilm formation by E. coli (*n* = 28), P. aeruginosa (*n* = 16), K. pneumoniae (*n* = 10), coagulase-negative staphylococci (CoNS), (*n* = 26), other Gram-negative (−) (*n* = 7), and Gram-positive (+) (*n* = 9) bacteria isolated from patients with bloodstream infections. Clinical isolates were classified as weak biofilm producers (WBPs) and strong biofilm producers (SBPs). All results are expressed as a percentage of strains with the specific biofilm-forming ability.

The assessment of biofilm formation (Fig. 1) revealed that weak biofilm producers (WBPs) (*n* = 68, 70.8%) were significantly (*P* < 0.0001) more abundant than strong biofilm producers (SBPs) (*n* = 28, 29.2%). The presence of SBPs was significantly (*P* = 0.002) attributable to Gram-negative strains. Specifically, SBPs were 7.1% (*n* = 2) for E. coli, 93.7% (*n* = 15) for P. aeruginosa, 50% (*n* = 5) for K. pneumoniae, and 3.8% (*n* = 1) for CoNS. Catheter-related BSIs (CRBSIs) were detected in 20.8% of episodes. In these cases, Gram-positive cocci represented the major fraction, with a 65% frequency of isolation (*n* = 13), while Gram-negative bacteria were found in 35% of cases (*n* = 7). SBPs were isolated in 30% (*n* = 6) of CRBSI episodes. However, we did not find any significant correlation between biofilm production and CRBSI. Also, we did not find any significant correlation between biofilm production and other demographic and clinical features at baseline, including diagnosed disease, disease status, comorbidities, and presence of MDR organism colonization. However, when we focused our attention on the clinical outcome by using a multivariate analysis, we found that the presence of SBP isolates was the only factor independently associated with end-organ disease (odds ratio [OR], 5.51; 95% confidence interval [CI], 1.34 to 22.67; *P* = 0.018) as well as a BSI sustained by P. aeruginosa (OR, 7.12; 95% CI, 1.27 to 40; *P* = 0.026). In particular, a BSI determined by SBP was significantly (OR, 5.35; 95% CI, 1.72 to 16.67; *P* = 0.004) associated with the increased risk of pneumonia occurrence.

**FIG 2:**
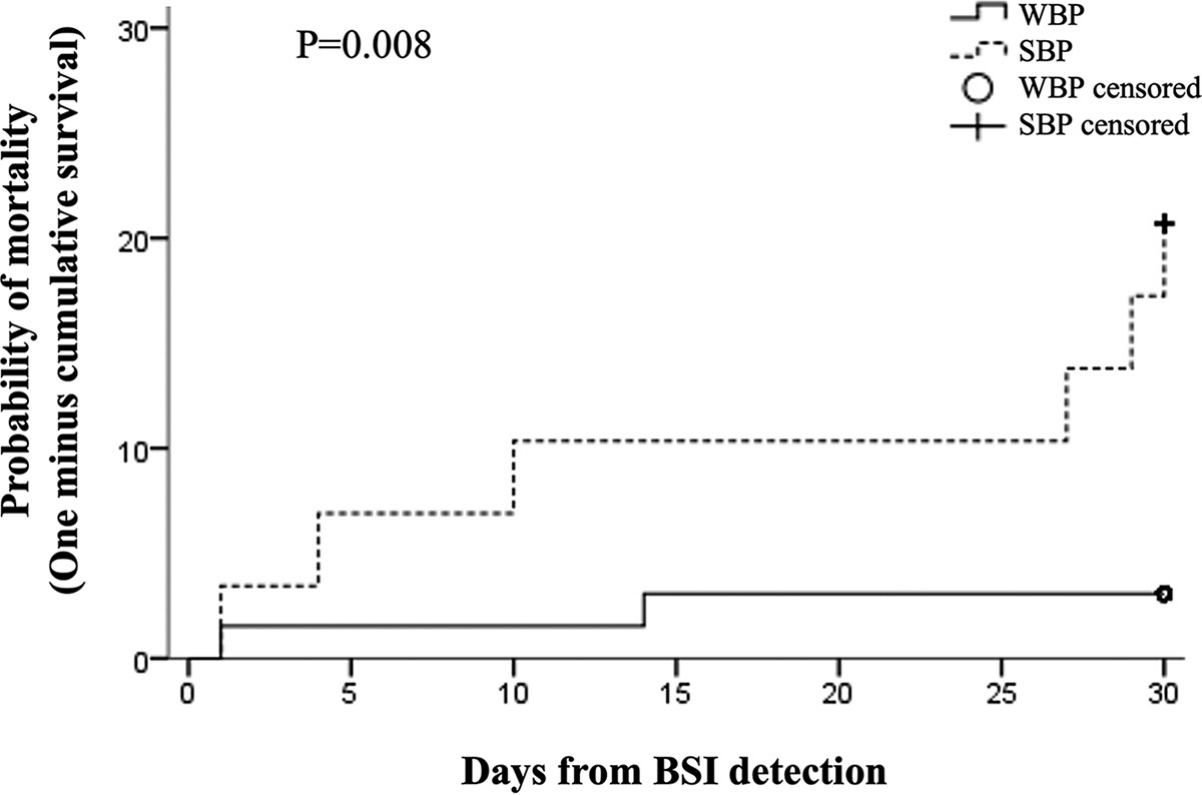
The actuarial probability of 30-day attributable mortality in patients with bloodstream infections (BSIs) according to strong biofilm producers (SBPs) versus weak biofilm producers (WBPs).

The overall attributable 30-day mortality rate was 8.3% (8/96). After a univariate analysis (Table 3), factors significantly associated with 30-day attributable mortality included the administration of antimicrobial prophylaxis (*P* = 0.035), the isolation of Gram-negative bacteria (*P* = 0.047), a BSI caused by P. aeruginosa (*P* = 0.027), MDR organisms (*P* < 0.0001) or by SBP strains (*P* = 0.010), the occurrence of a septic shock (*P* = 0.024), and end-organ disease (*P* = 0.005). As shown in Table 3, at multivariate analysis, SBP strains were found to be an independent risk factor associated with 30-day attributable mortality (hazard ratio, 10.47; 95% CI, 1.83 to 59.74; *P* = 0.008) together with an MDR phenotype (HR, 17.21; 95% CI, 2.04 to 145.52; *P* = 0.009) and the occurrence of a septic shock (HR, 9.65; 95% CI, 1.97 to 47.31; *P* = 0.005). Fig. 2 shows the actuarial probability of 30-day attributable mortality according to SBP versus WBP isolates.

**TABLE 3:** Univariate and multivariate analyses for 30-day attributable mortality^*a*^

Parameter	Univariate	Multivariate (forward selection)	
	*P* value	HR (95% CI)	*P* value
Age (continuous)	0.59		
Diagnosis (AL vs lymphoma vs myeloma)	0.51		
Comorbidities (yes vs no)	0.72		
Treatment phase (induction vs ASCT vs other)	0.59		
Disease status (onset vs CR vs PR vs refractory)	0.8		
Previous infections (yes vs no)	0.72		
Colonization by MDR organism (yes vs no)	0.64		
Antimicrobial prophylaxis (yes vs no)	0.035		
Grade ≥ 3 mucositis (yes vs no)	0.581		
Gram-negative vs Gram-positive BSI	0.047		
MDR vs non-MDR BSI	<0.001	17.21 (2.04–145.52)	0.009
Initial antimicrobial failure (yes vs no)	0.29		
P. aeruginosa vs other BSI	0.027		
SBP vs WBP	0.01	10.47 (1.83–59.74)	0.008
Septic shock (yes vs no)	0.024	9.65 (1.97–47.31)	0.005
End-organ disease (yes vs no)	0.005		

a AL, acute leukemia; ASCT, autologous hematopoietic stem cell transplant; CR, complete remission; PR, partial remission; MDR, multidrug resistant; BSI, bloodstream infection; WBP, weak biofilm producer; SBP, strong biofilm producer.

## DISCUSSION

We found a 13.4% prevalence of BSIs in HM patients requiring hospitalization in a hematology ward. This finding is in agreement with other studies performed in different settings and geographical areas (1, 9). Gram-negative bacteria were the most prevalent microbial species (63.5%) causing BSIs in our cohort of patients. These data are consistent with reports describing a shift of prevalence, during the last decade, from Gram-positive to Gram-negative bacteria among cancer patients with BSIs (7, 10). The reasons for this changing pattern are still unclear. The reduced use of fluoroquinolone prophylaxis has been proposed as one of the main reasons for the increase in Gram-negative bacteremia in neutropenic cancer patients (10). However, 73% of our patients received fluoroquinolone prophylaxis, suggesting that, at least in these cases, the increase of Gram-negative bacteria should be found elsewhere. Indeed, the increased frequency of Gram-negative bacteria causing BSIs in hematological settings may result from a specific host vulnerability to infections caused by the severe and prolonged neutropenia induced by chemotherapy (4, 10).

Among the Gram-negative bacteria isolated from BSIs, we found E. coli as the most frequent species (29.2%), followed by P. aeruginosa (16.7%), K. pneumoniae (10.4%), and A. baumannii (3.1%). CoNS were the most common Gram-positive bacteria (27.1%) isolated in our cohort of patients, followed by E. faecalis (3.1%), S. aureus (2.1%), and S. mitis (2.1%). These results are consistent with other large multicenter studies performed on HM patients in Italy and other countries (1, 7, 10). Of particular concern is the increase of Gram-negative bacteria worldwide, which corresponds to the emergence of drug-resistant strains, posing a high risk of failure of current prophylaxis and empirical treatment strategies (10). In our study, BSIs caused by MDR bacteria were 27% (*n* = 26) with a predominance of ESBL-producing *Enterobacteriaceae* (53.8%, *n* = 14). E. coli was the most prevalent bacteria (85.7%; *n* = 12) in the ESBL group, followed by K. pneumoniae (14.3%, *n* = 2). The development of infections caused by ESBL-producing *Enterobacteriaceae*, translocating from the intestinal tract into the bloodstream, has been attributed to the increased use of immunosuppressive agents and corticosteroids (11, 12). In fact, ESBL-producing *Enterobacteriaceae* represent the most frequent MDR bacteria isolated from BSIs in HM patients, with an increasing trend over the years (13, 14).

Notably, we found that WBPs causing BSIs were significantly more abundant than SBPs. This result is consistent with the notion that biofilms are a primary cause of chronic and recurrent infections but are associated only seldomly with acute infections (8). However, although less frequent than WBPs, when present, an SBP represents an independent risk factor for a most invasive infection and end-organ disease. This result suggests that once it enters the bloodstream, the ability to produce biofilm allows these bacteria to spread and colonize distant organs and tissues, leading to local metastatic infections. Infective endocarditis, pneumoniae, or septic arthritis are typical examples of infections caused by disseminating biofilm-producing bacteria to distant locations (8).

We observed a 30-day mortality rate of 8.3%, which is close to the overall rates reported previously by others (10, 15). Risk factors, such as the presence of SBPs (HR, 10.47; 95% CI, 1.83 to 59.74; *P* = 0.008), MDR organisms (HR, 17.21; 95% CI, 2.04 to 145.52; *P* = 0.009), and the occurrence of septic shock (HR, 9.65; 95% CI, 1.97 to 47.31; *P *= 0.005), were all associated independently with 30-day mortality in the multivariate analysis. A direct correlation between an infection sustained by MDR bacteria and poor clinical outcome represents a well-recognized risk factor, mainly due to ineffective empirical antimicrobial treatment in the first 24 hours of therapeutic management (1, 7). On the other hand, the presence of SBPs represents a novel microbial parameter for the clinical assessment of the risk of mortality. Few studies have evaluated the role of biofilm-producing bacteria in the clinical outcome of BSI. Data on different microbial species and clinical endpoints are heterogeneous since most studies so far have been focused on single bacterial species and/or performed in experimental animal models (16, 17), raising several controversies (18). Besides, the lack of a standardized protocol for biofilm characterization further limited a reliable comparative evaluation of the data (19). In the present study, the use of the BioFilm ring test (cBRT) allowed the early detection and profiling of biofilm-forming bacteria and provided a reliable and standardized tool for diagnosing and monitoring biofilm-associated infections within a routine clinical microbiology platform (20).

We found that biofilm production represents an important factor in the pathogenesis of P. aeruginosa and K. pneumoniae colonization/infection, with 93.7% and 50% of strains classified as SBPs, respectively. Biofilm formation around blood vessels is critical for P. aeruginosa and K. pneumoniae to promote bacteremia and systemic spread of the infection (21), and this may play a key role in BSI and secondary microbial localization with the end-organ disease. The variation in biofilm production observed among different bacterial species may depend on their differences in biofilm biochemical composition and the ensuing structure of the biofilm matrix (22). Indeed, we found that only a limited number of E. coli (7.1%) were SBPs, suggesting that biofilm production does not represent a key survival and pathogenic factor for E. coli. This finding is also supported by previous observations which failed to find an association between biofilm production and increased mortality rates in E. coli bacteremia (23). These results suggest that different microbial species may have adopted distinct strategies to exploit the shear stress of flowing blood and overcome host responses, ultimately leading to BSIs.

Biofilms readily grow on indwelling medical devices and have been reported to play a role in CRBSIs (8). However, in agreement with previous findings (18), we did not find any association between the presence of an intravascular device and the occurrence of BSIs caused by biofilm-producing isolates.

This study has some limitations; for example, the data were obtained from a single institution during a limited study period, and thus, the results may not be widely representative or generalizable. Another possible limitation derives from the static assessment of biofilm production by cBRT, which may underrepresent the biofilm-forming capability *in vivo* under the shear forces and dynamic flow conditions present in BSIs.

Overall, SBP bacteria raise the risk of end-organ disease in HM patients developing a BSI and that, together with an MDR phenotype, they can independently and significantly concur at increasing the risk of death. Thus, the characterization of microbial biofilms may provide key information for the diagnosis and therapeutic management of BSI and may help develop novel strategies to either eradicate or control harmful microbial biofilms.

## MATERIALS AND METHODS

### Patients.

We conducted a single-institution prospective cohort study in the Hematology and Stem Cell Transplant Unit, enrolling all consecutive adult patients (aged ≥18 years) affected by HMs and experiencing a BSI as detected from April 2016 through April 2019. All patients were screened at admission to assess for the presence of colonization by MDR isolates by rectal, nasal, pharyngeal, and urethral/genital swabs. All clinical and microbiological information was entered into a dedicated electronic database. Asymptomatic cases of BSIs seldomly detected by routine blood culture surveillance as well as polymicrobial infections were excluded from this study. The study was conducted according to the Helsinki declaration, and all enrolled patients signed informed consent. The ethics committee I.R.C.C.S. Lazio approved the study (protocol CE/1016/15-15 December 2015, trials registry no. 730/15).

### Definitions.

BSI was identified by single microbial isolation in one blood culture; two positive cultures were considered necessary for the diagnosis of coagulase-negative staphylococci or *Corynebacterium* BSI. Central venous catheter (CVC)-related BSIs were identified according to Mermel et al. (24). Sepsis and septic shock were identified according to Surviving Sepsis Campaign criteria (25). The Multinational Association of Supportive Care in Cancer (MASCC) score for febrile neutropenia risk was the reference for assessing the severity of the patient’s clinical presentation at first fever occurrence during neutropenia (26). Neutropenia was defined as an absolute neutrophil count (ANC) of <500 neutrophils/mcL at the onset of BSI; neutropenia was considered prolonged if the duration was 10 days or more and severe if ANC was <100 neutrophils/mcL. Antimicrobial prophylaxis with 500 mg ciprofloxacin twice daily was administered to all patients with an expected prolonged duration of neutropenia from April 2016 to January 2019, when such a “universal prophylaxis administration” approach was abrogated according to the local institutional authorities. Empiric broad-spectrum antimicrobial treatment was started at the onset of fever in all neutropenic patients according to the local protocols and international guidelines (27, 28). Pneumonia was documented and confirmed by a bronchoalveolar lavage fluid analysis (29), and soft tissue infection was documented by computed tomography (CT) scan and confirmed by cultural exam of a pathological tissue biopsy specimen.

### Microbiology.

Blood samples were collected by inoculating 10 ml in aerobic (BD-Bactec plus Aerobic/F) and anaerobic (BD-Bactec plus Anaerobic/F) culture vials and incubating in the BD-Bactec FX40 automated instrument system (Becton, Dickinson Microbiology Systems) for 5 days. Samples were collected from a total of 96 adult patients affected by HMs with a BSI. Bacterial identification and antimicrobial susceptibility testing were performed as previously described (20, 30). Multidrug-resistant (MDR) bacteria were defined according to the European Centre for Disease Prevention and Control (ECDC) criteria (https://www.ecdc.europa.eu/en).

Biofilm production was assessed by the clinical BioFilm ring test (cBRT) (Biofilm Control, Saint Beauzire, France) as previously described (20). Each strain was analyzed in duplicate, and experiments were repeated at least two times.

### Statistics.

All variables were summarized with descriptive statistics and tested for normality. Comparisons between continuous variables were carried out with the Student’s *t* test or the Mann-Whitney U test, when appropriate, while categorical variables were tested using the χ^2^ or two-tailed Fisher’s exact test, when appropriate. Thirty-day attributable mortality analysis was performed by using the Cox proportional hazard regression model. Attributable mortality was defined as the time elapsed from BSI detection to the patient’s death within 30 days of observation after BSI diagnosis. The Hazard risks and their relative 95% confidence intervals (95% CIs) were estimated for each variable using the Cox univariate model and adopting the most suitable prognostic modality as a referent group. A multivariate Cox proportional hazard model was then conducted considering the variables significant at univariate analysis using stepwise regression (forward selection). Enter limit and remove limit were set at *P* values of 0.05 and 0.10, respectively. A *P* value of <0.05 was considered statistically significant. Statistical analyses were performed using SPSS software (SPSS version 21, SPSS Inc., Chicago, IL, USA).
